# Compensatory substitutions in the HCV NS3/4A protease cleavage sites are not observed in patients treated unsuccessfully with telaprevir combination treatment

**DOI:** 10.1186/1743-422X-9-147

**Published:** 2012-08-06

**Authors:** James C Sullivan, Eileen Z Zhang, Doug J Bartels, Ann Tigges, Jennifer L Dorrian, Ann D Kwong, Tara L Kieffer

**Affiliations:** 1 Vertex Pharmaceuticals Incorporated, 130 Waverly Street, Cambridge, MA 02139, USA

**Keywords:** Directional selection, Fitness, INCIVEK, Telaprevir

## Abstract

**Background:**

Development of compensatory mutations within the HIV p7/p1 and p1/p6 protease cleavage site region has been observed in HIV-infected patients treated with protease inhibitors. Mechanisms of fitness compensation may occur in HCV populations upon treatment of HCV protease inhibitors as well.

**Findings:**

In this study, we investigated whether substitutions in protease cleavage site regions of HCV occur in response to a treatment regimen containing the NS3/4A protease inhibitor telaprevir (TVR). Evaluation of viral populations from 569 patients prior to treatment showed that the four NS3/4A cleavage sites were well conserved. Few changes in the cleavage site regions were observed in the 159 patients who failed TVR combination treatment, and no residues displayed evidence of directional selection after the acquisition of TVR-resistance.

**Conclusions:**

Cleavage site mutations did not occur after treatment with the HCV protease inhibitor telaprevir.

## Findings

Protease inhibitors (PI) are part of the current treatment regimens for both HIV and HCV infections. During treatment, levels of wild-type virus sensitive to PI are reduced, but PI-resistant variants may be selected. These resistant variants may have a decreased binding affinity for the PI, but also typically have a lower replicative capacity, likely due to a decreased processing efficiency.

During HIV PI treatment, in addition to the primary resistant variants observed in the catalytic site of the protease, variants have been observed within the protease substrates of p7/p1 and p1/p6 of the Gag-Pol polyprotein. These *cis* mutations in the protease substrates compensate the protease impairment caused by the PI-resistant mutations. Such mechanisms improve the protease and substrate interaction and the fitness of viral genomes carrying PI-resistant variants. Additionally, cleavage site mutations may directly contribute to resistance to PIs, potentially by improving the cleavage of the Gag region. Indeed, cleavage site variants have been observed in the absence of primary PI resistance mutations [1-4].

Phase 3 clinical studies investigating TVR combination treatment demonstrated significant improvement of sustained viral response (SVR) rates compared to peginterferon alfa-2a and ribavirin alone (PR) in HCV genotype 1 infected patients [5,6]. In a subset of patients who failed to achieve SVR after a TVR-containing regimen, TVR-resistant variants were observed in the NS3 protease, with the variants V36M and R155K observed most commonly in subtype 1a infections and variants V36A, T54A, and A156S/T observed most commonly in subtype 1b infections [7-9]. These resistant variants were less fit than wild-type virus in vitro and in vivo [10,11]. In this study, we sought to determine if cleavage site mutations also occur, either in the presence or absence of catalytic site mutations in the HCV NS3/4A protease, to improve the processing capacity of the protease as observed during HIV infection treatment.

Our study included PI treatment-naïve patients who had chronic, genotype 1 HCV infection and were enrolled in Phase 2 clinical studies of TVR (PROVE1 and PROVE2) [12,13]. These studies were conducted in full compliance with the guidelines of Good Clinical Practice and of the World Medical Assembly Declaration of Helsinki. The protocols and informed consent forms of these studies were approved by an independent or institutional review board at each institution, and written informed consent for publication of this analysis was provided by all patients prior to participating in study-related activities. A copy of written consent is available for review by the Editor-in-Chief of this journal. Patients received various combinations of TVR, peginterferon alfa-2a (40 kDa), and/or ribavirin as described in detail in McHutchison *et al.*[12] and Hézode *et al*. [13]. Population sequence analysis of the HCV NS3/4A protease and protease cleavage sites was performed before, during, and after treatment as described previously [14].

The HCV serine protease NS3/4A cleaves four downstream substrates during HCV polyprotein translation, which is essential for viral replication. Autocleavage of the C terminus of NS3 from the N terminus of NS4 occurs in *cis* during translation of the nascent polyprotein chain, and is followed by cleavage of NS5A-NS5B, NS4A-NS4B, and NS4B-NS5A [15]. In this study, sequence data were available from 569 patients before treatment. Data from these patients were used to determine the expected frequency of amino acid variants at each position within 10 residues on either side (P10 to P10’) of each cleavage site in the non-structural region.

Patients who failed to achieve SVR after TVR combination treatment had received an average of 10.9 weeks of TVR (± 2.75; standard deviation; n = 116). After treatment failure, an average of 3.7 follow-up visits was available for assessment of viral populations from each patient, allowing an average of 7.7 weeks of follow-up (Additional file 1: Table S1). During each assessment, viral population sequencing was performed for the entire non-structural region (PROVE1 patients; providing sequence data for all four cleavage sites), and for the NS3-4A genomic region (PROVE2 patients; providing sequence data for two cleavage sites; Table 1). Additionally, sequence data at all four cleavage sites were available from 43 patients who had failed to achieve SVR in the PR control arm of PROVE1, and from 28 patients at two cleavage sites (NS3-NS4A and NS4A-NS4B) from the control arm of PROVE2.

**Table 1:** Sequence Dataset

	**TVR-Treatment Arms**^***a***^			**PR-Treatment Arms**^***b***^		
**Cleavage Site**	**1a**	**1b**	**Total**	**1a**	**1b**	**Total**
**NS3-NS4A**	71	45	116	26	17	43
**NS4A-NS4B**	71	45	116	26	17	43
**NS4B-NS5A**^***c***^	34	9	43	19	9	28
**NS5A-NS5B**^***c***^	34	9	43	19	9	28

^*a*^ This group includes patients who failed to achieve SVR after TVR combination treatment.

^*b*^ This group includes patients who failed to achieve SVR after the control PR treatment (*i.e.*, patients who did not receive TVR).

^*c*^ The NS5A-NS5B genomic region was not sequenced for PROVE2.

We calculated the probability of the observed frequency (termed *F*) of each residue at each position within patients who had failed to achieve SVR after TVR combination treatment, given the frequency of that residue in a TVR-naïve dataset (termed *f*), with the neutral rate of change assumed to fit a Poisson distribution and with type I error controlled using a Bonferroni correction. The expected, *i.e., *treatment-naïve frequency, was derived from the baseline frequency with which a variant was observed in a large, semi-independent dataset that pooled pre-treatment data from all Phase 2 and 3 studies of telaprevir. Only those combinations of site and amino acid wherein *F > f* (*i.e.*, changes enriched in the TVR-treatment arms relative to expectation) were analyzed statistically.

**Figure 1:**
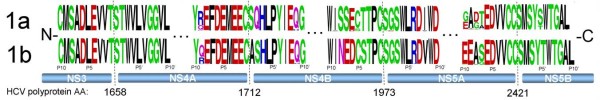
**Baseline conservation of cleavage sites.** Sequence logos (Schnieder ref [18], Crooks ref [19]) were generated from baseline (pre-treatment) HCV sequence. Dashed lines indicate cleavage points along the HCV polyprotein, with the amino acid numbering below the cleavage site referencing HCV genotype 1a reference strain H77 (Genbank accession NC_004102). Cartoons of proteins are not drawn to scale. (*Sample size for each sequence logo— NS3-NS4A and NS4A-NS4B: 1a = 327 patients, 1b = 242 patients; NS4B-NS5A and NS5A-NS5B: 1a = 219 patients, 1b = 108 patients*).

Before treatment, the 20 amino acid residues surrounding the four HCV NS3/4A cleavage sites were highly conserved. Consistent with previous reports [16], our data indicate that in all four NS3/4A substrates, S and A predominate at the P1’ site, C or T predominate at the P1 site, and the acidic residues E and D predominate at the P6 site. Despite the high degree of conservation within subtype 1a and 1b populations, there were characteristic differences between subtypes 1a and 1b within the region surrounding cleavage sites (Figure 1). That each of the four cleavage site regions is well conserved implies that they are under strong stabilizing selection, and that the translated peptide they encode should be stable in established populations. This was supported by the analysis of 43 patients who had failed to respond to PR treatment in the control arm of the studies. Indeed, during treatment, only 1.1% (31/2840) of residues changed in 142 cleavage sites analyzed (≤ 4 cleavage sites analyzed per patient; Table 1). Similarly, only 0.52% (33/6360) of the residues changed in 318 cleavage site regions analyzed from 116 patients who had failed to respond to TVR combination treatment (≤ 4 cleavage sites analyzed per patient; Table 1).

Of the 116 patients analyzed during and after failing to respond to TVR combination treatment, 105 had detectable TVR-resistant variants within NS3 (Additional file 1: Table S1). In the TVR combination treatment arms, 10 variants in the cleavage site region were observed after treatment where the variant was present at a greater frequency after treatment failure than before treatment. All of these changes were unique to a single patient (Table 2). Not surprisingly given the rare nature of these changes, none of the changes reached statistical thresholds of significance, regardless of whether the analysis utilized subtype-specific or pooled genotype 1 treatment-naïve frequencies.

**Table 2:** Probability of occurrence of changes in TVR combination treatment arms, based on a TVR-naïve dataset

			**TVR-naïve dataset**			**TVR-resistant dataset**			
**Position**	**Site**	**AA**	**x**^**†**^	**X**^‡^	***f*(%)**	**n**^**†**^	**N**^‡^	***F*(%)**	***Prob (F|f)***
NS3 625	P7	T	0	3488	0.00	1	116	0.86	0.0322^*^
NS4A 9	P9’	I	4	3488	0.11	1	116	0.86	0.1165
NS4A 45	P10	H	0	3488	0.00	1	116	0.86	0.0322*
NS4B 2	P2’	L	5	3469	0.14	1	116	0.86	0.1415
NS4B 2	P2’	A	22	3469	0.63	1	116	0.86	0.3525
NS4B 4	P4’	F	5	3483	0.14	1	116	0.86	0.1410
NS4B 254	P8	G	2	776	0.26	1	43	2.33	0.0992
NS5A 2	P2’	S	2	772	0.26	1	43	2.33	0.0997
NS5A 440	P9	G	8	765	1.05	1	43	2.33	0.2868
NS5A 441	P8	P	0	761	0.00	1	43	2.33	0.0534*
NS5A 441	P8	T	3	761	0.39	2	43	4.65	0.0121*
NS5B 7	P7’	S	0	777	0.00	1	43	2.33	0.0524^*^

^**†**^* n,x: denote the number of times each residue was observed in each dataset; *^**‡**^* N,X: indicate the sample size for each dataset.*

* prob- values associated with any residues which are not observed in the TVR-naïve dataset (i.e., *x = 0) are marked with an asterisk. The neutrally expected frequency of these amino acids at these positions can only be defined as < 1/X; therefore calculations for each of these positions assume 1 occurrence of the residue in the TVR-naïve dataset. Probability values for these residues should therefore be interpreted as upper bounds.*

Given the lack of statistically significant enrichment of any variants in TV R treatment arms for each of the residues analyzed, those changes in which the derived residue in the TVR-naïve dataset is rare or absent can most likely be explained by a genetic drift through bottleneck events. During TVR combination treatment, the inhibition of viral production leads to a rapid drop in serum HCV RNA. Due to this loss of wild-type virus, the low baseline levels of TVR-resistant variants are enriched in the viral population. These pre-existing resistant variants will likely carry numerous nucleotide changes, relative to the dominant quasispecies prior to treatment, some of which will be coding changes. In this way, neutral or even deleterious changes can become fixed within HCV populations due to the significant TVR-imparted selective pressure.

To further assess this hypothesis, we calculated the difference between the rate of non-synonymous (dN) to synonymous (dS) nucleotide changes by codon in alignments of the cleavage sites that included the pretreatment and all on-treatment time points [17]. In this way, the relative rates of synonymous and non-synonymous nucleotide substitutions would capture the time period during which the viral population would be expected to undergo a directional selective sweep because of the selective pressure of the inhibitor. Under this model, a normalized dN -dS difference that is significantly greater than 0 suggests that directional selective pressure is acting on the codon, whereas values less than 0 suggest stabilizing selection. No codon had evidence for directional selection during treatment (median p-value: 0.99938; range: 0.39, 1), and indeed, the maximum difference between dN and dS was only 0.37 (median: -1.3; range: -6.75, 0.37). These data support that interpretation that HCV cleavage site regions within the non-structural region of the poly-protein are under strong stabilizing selection, and that changes observed during treatment reflect stochastic events rather than directional selection.

In this study, we assessed whether changes in any of the four NS3/4A protease cleavage sites were a significant mechanism of viral fitness improvement during TVR treatment in the presence or absence of PI-resistant variants. Our data showed that no amino acid in the 20 residues surrounding the cleavage sites presented evidence of primary resistance or compensatory mutations.

## Abbreviations

HIV: Human immunodeficiency virus; HCV: Hepatitis C virus; WT: Wild-type; TVR: Telaprevir; P: Peginterferon alfa-2a; RBV or R: Ribavirin; PR: Peginterferon alfa-2a and ribavirin; PI: Protease inhibitor.

## Competing interests

This work was supported by Vertex Pharmaceuticals Incorporated, Cambridge MA, USA. All authors are employees and stock holders of Vertex Pharmaceuticals Incorporated, Cambridge MA, USA.

## Authors’ contributions

TLK, ADK conceived of the experiment; JCS, EZZ, DJB, TLK designed the experiment, EZZ, JLD executed experimental design, AT provided computational support, JCS, DJB analyzed data; JCS, EZZ, DJB, AT, JLD, ADK and TLK authored the document. All authors read and approved the final manuscript.

## Authors’ information

JCS, EZZ, DJB, AT, JLD, and TLK are employees of Vertex Pharmaceuticals Incorporated, as was ADK at the time this work was performed.

## Supplementary Material

Additional file 1 **Table S1.** Individual patient data in the TVR combination treatment arms. Click here for file
