# Supplementary material for: Compensatory substitutions in the HCV NS3/4A protease cleavage sites are not observed in patients treated unsuccessfully with telaprevir combination treatment
Source: Virol J. 2012 Aug 6;9:147. doi: 10.1186/1743-422X-9-147 (PMC3499439; doi:10.1186/1743-422X-9-147)
Supplement: Additional file 1 — Table S1. Individual patient data in the TVR combination treatment arms. [file 1743-422X-9-147-S1.doc]

Supplemental Table 1. Individual patient data in the TVR combination treatment arms

| **Study** | **Arm^1^** | **Sub-**  **type** | **TVR**  **(weeks)^2^** | **Log**  **(BVL)^3^** | **Log (Nadir)^4^** | **Bktgh / relapse (weeks)^5^** | **Log (Post Nadir)^6^** | **# Post Nadir^7^** | **Last Time – Bktgh /relapse^8^** | **Log (Last VL)^9^** | **Associated TVR-Resistant**  **Mutations ^10^** |
| --- | --- | --- | --- | --- | --- | --- | --- | --- | --- | --- | --- |
| PROVE2 | TVR/PR 12w + None | 1a | 11.86 | 6.84 | 3.67 | 3.86 | 3.87 | 12 | 48.14 | 6.21 | 155 |
| PROVE2 | TVR/PR 12w + None | 1a | 12.00 | 6.77 | 2.89 | 3.00 | 4.51 | 7 | 10.86 | 6.67 | 36,155 |
| PROVE2 | TVR/PR 12w + None | 1a | 12.00 | 6.62 | 0.70 | 16.00 | 5.83 | 1 | 0.00 | 5.83 | 155 |
| PROVE2 | TVR/PR 12w + None | 1a | 11.86 | 6.12 | 0.70 | 19.86 | 4.64 | 2 | 4.00 | 5.97 | 155 |
| PROVE2 | TVR/PR 12w + None | 1a | 12.00 | 7.17 | 1.30 | 3.14 | 1.49 | 13 | 31.86 | 6.49 | 36,155 |
| PROVE2 | TVR/PR 12w + None | 1a | 12.00 | 5.94 | 1.30 | 3.00 | 3.23 | 7 | 11.00 | 5.58 | 36,155 |
| PROVE2 | TVR/PR 12w + None | 1a | 12.00 | 6.60 | 0.70 | 20.00 | 5.50 | 1 | 0.00 | 5.50 | 36,155 |
| PROVE1 | TVR/PR 12w + None | 1a | 3.14 | 7.23 | 0.70 | 29.14 | 7.19 | 1 | 0.00 | 7.19 | 36,155 |
| PROVE2 | TVR/PR 12w + None | 1a | 12.00 | 6.55 | 2.43 | 2.00 | 4.48 | 11 | 24.00 | 5.78 | 36,155 |
| PROVE2 | TVR/PR 12w + None | 1a | 12.00 | 7.04 | 0.70 | 19.00 | 6.93 | 2 | 1.57 | 6.92 | 155 |
| PROVE2 | TVR/PR 12w + None | 1a | 11.86 | 6.46 | 3.21 | 1.86 | 3.55 | 12 | 12.14 | 6.34 | 36,155 |
| PROVE2 | TVR/PR 12w + None | 1a | 12.00 | 6.81 | 1.51 | 4.14 | 2.36 | 7 | 12.00 | 5.29 | 36,155 |
| PROVE2 | TVR/PR 12w + None | 1a | 12.00 | 6.56 | 0.70 | 15.71 | 1.30 | 2 | 4.00 | 6.13 | 36,155 |
| PROVE1 | TVR/PR 12w + None | 1a | 12.00 | 7.18 | 0.70 | 45.43 | 3.41 | 2 | 1.43 | 5.62 | 36,155 |
| PROVE2 | TVR/PR 12w + None | 1a | 12.00 | 6.11 | 3.09 | 0.58 | 4.2 | 11 | 19.57 | 2.00 | 36,155 |
| PROVE2 | TVR/PR 12w + None | 1a | 12.29 | 6.08 | 0.70 | 20.29 | 5.88 | 3 | 16.00 | 5.73 | 36,155 |
| PROVE2 | TVR/PR 12w + None | 1a | 12.00 | 6.69 | 0.70 | 19.86 | 4.88 | 1 | 0.00 | 4.88 | 36 |
| PROVE1 | TVR/PR 12w + None | 1a | 12.00 | 6.29 | 0.70 | 21.14 | 1.48 | 2 | 1.00 | 3.33 | 155 |
| PROVE2 | TVR/PR 12w + None | 1a | 12.00 | 7.19 | 2.13 | 6.00 | 3.84 | 4 | 10.00 | 0.70 | 36,54,155 |
| PROVE2 | TVR/PR 12w + None | 1a | 12.00 | 6.68 | 3.78 | 1.00 | 4.30 | 12 | 13.00 | 6.62 | 155 |
| PROVE1 | TVR/PR 12w + None | 1a | 12.00 | 5.94 | 0.70 | 23.14 | 1.30 | 3 | 3.29 | 3.80 | 155 |
| PROVE1 | TVR/PR 12w + None | 1a | 12.00 | 6.24 | 0.70 | 49.43 | 1.59 | 1 | 0.00 | 1.59 | 36,155 |
| PROVE2 | TVR/PR 12w + None | 1a | 12.00 | 6.62 | 0.70 | 8.00 | 1.30 | 5 | 6.00 | 4.19 | 36,155 |
| PROVE2 | TVR/PR 12w + None | 1a | 11.57 | 6.34 | 0.70 | 14.00 | 1.30 | 3 | 3.29 | 6.19 | 36,155 |
| PROVE1 | TVR/PR 12w + None | 1a | 12.14 | 7.05 | 0.70 | 21.14 | 1.30 | 4 | 3.00 | 5.93 | 155 |
| PROVE2 | TVR/PR 12w + None | 1a | 12.00 | 7.37 | 0.70 | 19.86 | 1.30 | 2 | 3.86 | 6.42 | 155 |
| PROVE1 | TVR/PR 12w + PR 12w | 1a | 10.71 | 6.48 | 2.25 | 2.00 | 2.53 | 7 | 9.43 | 5.76 | 36,155 |
| PROVE1 | TVR/PR 12w + PR 12w | 1a | 12.00 | 7.19 | 0.70 | 27.86 | 6.07 | 2 | 1.14 | 6.29 | 155 |
| PROVE2 | TVR/PR 12w + PR 12w | 1a | 11.57 | 5.97 | 0.70 | 16.43 | 4.46 | 1 | 0.00 | 4.46 | 36,155 |
| PROVE1 | TVR/PR 12w + PR 12w | 1a | 4.86 | 7.40 | 0.70 | 35.86 | 6.31 | 1 | 0.00 | 6.31 | None |
| PROVE2 | TVR/PR 12w + PR 12w | 1a | 11.86 | 6.89 | 0.70 | 15.57 | 5.82 | 1 | 0.00 | 5.82 | 36,155 |
| PROVE2 | TVR/PR 12w + PR 12w | 1a | 12.00 | 6.94 | 0.70 | 14.43 | 2.56 | 2 | 1.57 | 6.04 | 155 |
| PROVE1 | TVR/PR 12w + PR 12w | 1a | 11.86 | 6.68 | 0.70 | 31.43 | 3.75 | 1 | 0.00 | 3.75 | 155 |
| PROVE1 | TVR/PR 12w + PR 12w | 1a | 8.00 | 7.05 | 1.30 | 36.00 | 2.79 | 3 | 14.00 | 6.22 | 36 |
| PROVE2 | TVR/PR 12w + PR 12w | 1a | 12.00 | 6.32 | 0.70 | 20.00 | 5.50 | 1 | 0.00 | 5.50 | 155 |
| PROVE1 | TVR/PR 12w + PR 12w | 1a | 10.86 | 6.51 | 6.45 | 0.43 | 5.73 | 7 | 7.57 | 3.70 | 36,54,155,156 |
| PROVE1 | TVR/PR 12w + PR 12w | 1a | 8.57 | 6.92 | 0.70 | 13.00 | 6.25 | 1 | 0.00 | 6.25 | 36,155 |
| PROVE1 | TVR/PR 12w + PR 12w | 1a | 12.00 | 5.81 | 0.70 | 10.00 | 1.30 | 6 | 23.00 | 5.59 | 36,155 |
| PROVE2 | TVR/PR 12w + PR 12w | 1a | 12.00 | 6.11 | 0.70 | 20.43 | 5.71 | 2 | 3.57 | 5.87 | 36,155 |
| PROVE1 | TVR/PR 12w + PR 12w | 1a | 11.71 | 6.96 | 0.70 | 3.86 | 1.30 | 9 | 35.14 | 6.68 | 36,155 |
| PROVE2 | TVR/PR 12w + PR 12w | 1a | 12.14 | 6.52 | 0.70 | 16.00 | 4.30 | 1 | 0.00 | 4.30 | 156 |
| PROVE1 | TVR/PR 12w + PR 12w | 1a | 12.14 | 7.00 | 0.70 | 24.00 | 2.11 | 3 | 2.00 | 4.58 | 155 |
| PROVE1 | TVR/PR 12w + PR 12w | 1a | 11.86 | 6.36 | 0.70 | 15.86 | 2.20 | 4 | 9.00 | 6.37 | 36,155 |
| PROVE1 | TVR/PR 12w + PR 12w | 1a | 5.43 | 7.09 | 0.70 | 36.86 | 6.57 | 1 | 0.00 | 6.57 | 155 |
| PROVE2 | TVR/PR 12w + PR 12w | 1a | 12.00 | 7.08 | 0.70 | 24.00 | 5.13 | 1 | 0.00 | 5.13 | 155 |
| PROVE2 | TVR/PR 12w + PR 12w | 1a | 12.00 | 6.78 | 0.70 | 24.00 | 3.45 | 1 | 0.00 | 3.45 | 156 |
| PROVE1 | TVR/PR 12w + PR 12w | 1a | 10.29 | 7.04 | 0.70 | 12.00 | 1.30 | 2 | 10.14 | 5.98 | 36,155 |
| PROVE1 | TVR/PR 12w + PR 12w | 1a | 6.43 | 6.75 | 2.65 | 1.00 | 3.53 | 18 | 49.00 | 6.19 | 36,155,156 |
| PROVE1 | TVR/PR 12w + PR 12w | 1a | 12.00 | 6.92 | 0.70 | 56.00 | 4.95 | 1 | 0.00 | 4.95 | 36,155 |
| PROVE1 | TVR/PR 12w + PR 12w | 1a | 9.00 | 6.39 | 0.70 | 11.00 | 3.40 | 2 | 21.71 | 5.99 | 36,155 |
| PROVE1 | TVR/PR 12w + PR 36w | 1a | 11.86 | 6.64 | 0.70 | 51.00 | 4.89 | 2 | 8.00 | 6.49 | 36 |
| PROVE1 | TVR/PR 12w + PR 36w | 1a | 0.57 | 6.24 | 2.70 | 1.00 | 4.80 | 4 | 5.00 | 5.68 | None |
| PROVE2 | TVR/PR 12w + PR 36w | 1a | 12.00 | 6.01 | 5.41 | 0.43 | 4.63 | 11 | 19.43 | 2.46 | 36,54,155 |
| PROVE1 | TVR/PR 12w + PR 36w | 1a | 4.86 | 7.13 | 0.70 | 52.00 | 5.98 | 1 | 0.00 | 5.98 | 36,155 |
| PROVE2 | TVR/PR 12w + PR 36w | 1a | 12.00 | 6.93 | 3.09 | 1.14 | 3.18 | 10 | 17.00 | 5.71 | 36,155,156 |
| PROVE1 | TVR/PR 12w + PR 36w | 1a | 12.00 | 7.00 | 0.70 | 24.14 | 2.16 | 5 | 26.00 | 6.06 | 54,155 |
| PROVE1 | TVR/PR 12w + PR 36w | 1a | 12.00 | 6.17 | 0.70 | 26.43 | 1.64 | 4 | 25.86 | 6.35 | 54,155 |
| PROVE1 | TVR/PR 12w + PR 36w | 1a | 11.86 | 6.73 | 0.70 | 24.00 | 1.30 | 4 | 7.86 | 4.20 | 36,155 |
| PROVE2 | TVR/PR 12w + PR 36w | 1a | 12.00 | 6.98 | 0.70 | 25.00 | 1.30 | 3 | 2.86 | 6.21 | 36,155 |
| PROVE1 | TVR/PR 12w + PR 36w | 1a | 12.00 | 6.58 | 0.70 | 21.14 | 6.50 | 1 | 0.00 | 6.50 | None |
| PROVE1 | TVR/PR 12w + PR 36w | 1a | 10.29 | 6.79 | 0.70 | 19.00 | 6.74 | 2 | 6.57 | 6.44 | None |
| PROVE2 | TVR/PR 12w + PR 36w | 1a | 12.00 | 6.77 | 0.70 | 28.00 | 2.73 | 2 | 4.00 | 5.94 | 36,155 |
| PROVE1 | TVR/PR 12w + PR 36w | 1a | 12.14 | 5.98 | 0.70 | 36.43 | 4.64 | 1 | 0.00 | 4.64 | 36 |
| PROVE1 | TVR/PR 12w + PR 36w | 1a | 12.00 | 6.86 | 0.70 | 12.00 | 1.56 | 8 | 17.29 | 6.12 | 36,155 |
| PROVE1 | TVR/PR 12w + PR 36w | 1a | 10.71 | 6.68 | 0.70 | 55.71 | 5.33 | 1 | 0.00 | 5.33 | 36,155 |
| PROVE1 | TVR/PR 12w + PR 36w | 1a | 11.57 | 7.01 | 0.70 | 22.00 | 6.57 | 3 | 4.00 | 6.85 | 36,155 |
| PROVE1 | TVR/PR 12w + PR 36w | 1a | 0.86 | 6.77 | 2.53 | 2.57 | 6.12 | 1 | 0.00 | 6.12 | None |
| PROVE1 | TVR/PR 12w + PR 36w | 1a | 12.14 | 6.87 | 0.70 | 16.14 | 1.30 | 2 | 3.00 | 5.78 | 36,155 |
| PROVE2 | TVR/PR 12w + PR 36w | 1a | 12.00 | 7.12 | 3.48 | 2.00 | 3.60 | 12 | 28.29 | 6.12 | 36,155,156 |
| PROVE2 | TVR/PR 12w + PR 36w | 1a | 12.00 | 6.91 | 0.70 | 25.14 | 1.62 | 2 | 1.00 | 4.10 | 36,155 |
| PROVE2 | TVR/PR 12w + PR 36w | 1a | 0.86 | 6.64 | 1.72 | 2.00 | 3.81 | 3 | 2.00 | 3.08 | None |
| PROVE2 | TVR/PR 12w + None | 1b | 12.00 | 6.45 | 2.79 | 2.00 | 4.56 | 11 | 24.00 | 6.00 | 54,155,156 |
| PROVE2 | TVR/PR 12w + None | 1b | 12.00 | 6.86 | 1.30 | 6.00 | 3.01 | 8 | 30.43 | 6.56 | 54 |
| PROVE1 | TVR/PR 12w + None | 1b | 12.00 | 7.06 | 0.70 | 14.86 | 2.42 | 6 | 5.00 | 6.19 | 54 |
| PROVE2 | TVR/PR 12w + None | 1b | 12.00 | 5.84 | 0.70 | 14.00 | 1.30 | 3 | 5.86 | 5.75 | 36,54 |
| PROVE2 | TVR/PR 12w + None | 1b | 12.00 | 6.42 | 2.36 | 7.00 | 3.70 | 8 | 16.86 | 0.70 | 36,54,156 |
| PROVE2 | TVR/PR 12w + None | 1b | 11.86 | 6.85 | 0.70 | 14.00 | 1.30 | 4 | 10.14 | 6.15 | 54 |
| PROVE2 | TVR/PR 12w + None | 1b | 11.86 | 5.38 | 0.70 | 15.86 | 4.47 | 2 | 4.00 | 5.84 | 36 |
| PROVE2 | TVR/PR 12w + None | 1b | 12.00 | 5.73 | 0.70 | 12.00 | 1.30 | 3 | 2.00 | 3.05 | 54,156 |
| PROVE1 | TVR/PR 12w + None | 1b | 1.00 | 6.26 | 1.30 | 2.86 | 5.65 | 1 | 0.00 | 5.65 |  |
| PROVE2 | TVR/PR 12w + None | 1b | 12.14 | 6.97 | 0.70 | 21.57 | 1.30 | 2 | 4.00 | 5.61 | 54 |
| PROVE2 | TVR/PR 12w + None | 1b | 12.00 | 5.71 | 1.30 | 2.86 | 2.41 | 7 | 13.14 | 5.34 | 54,156 |
| PROVE1 | TVR/PR 12w + None | 1b | 3.00 | 6.33 | 0.70 | 7.29 | 3.86 | 1 | 0.00 | 3.86 |  |
| PROVE2 | TVR/PR 12w + None | 1b | 12.00 | 7.18 | 0.70 | 16.00 | 3.41 | 2 | 4.00 | 6.10 | 54 |
| PROVE2 | TVR/PR 12w + None | 1b | 12.00 | 6.84 | 0.70 | 50.57 | 2.42 | 1 | 0.00 | 2.42 | 36 |
| PROVE2 | TVR/PR 12w + None | 1b | 11.86 | 5.60 | 0.70 | 19.71 | 5.37 | 2 | 5.71 | 5.44 | 54 |
| PROVE2 | TVR/PR 12w + None | 1b | 12.00 | 6.49 | 0.70 | 15.86 | 2.05 | 2 | 4.00 | 5.49 | 156 |
| PROVE2 | TVR/PR 12w + None | 1b | 12.00 | 6.28 | 1.30 | 6.00 | 1.76 | 9 | 23.00 | 6.11 | 54,156 |
| PROVE2 | TVR/PR 12w + None | 1b | 8.43 | 5.78 | 0.70 | 44.71 | 6.56 | 1 | 0.00 | 6.56 | None |
| PROVE2 | TVR/PR 12w + None | 1b | 11.86 | 6.27 | 0.70 | 3.00 | 1.30 | 7 | 10.86 | 3.95 | 36,156 |
| PROVE2 | TVR/PR 12w + PR 12w | 1b | 12.00 | 7.10 | 0.70 | 16.00 | 1.30 | 4 | 12.00 | 6.16 | 36,54 |
| PROVE2 | TVR/PR 12w + PR 12w | 1b | 12.00 | 6.68 |  | 24.14 | 1.30 | 1 | 0.00 | 1.30 | 156 |
| PROVE2 | TVR/PR 12w + PR 12w | 1b | 11.00 | 6.19 | 0.70 | 18.57 | 4.88 | 1 | 0.00 | 4.88 | 54,156 |
| PROVE1 | TVR/PR 12w + PR 12w | 1b | 12.00 | 7.26 | 0.70 | 34.86 | 6.02 | 1 | 0.00 | 6.02 | None |
| PROVE1 | TVR/PR 12w + PR 12w | 1b | 9.00 | 6.65 | 0.70 | 32.57 | 7.26 | 1 | 0.00 | 7.26 | None |
| PROVE1 | TVR/PR 12w + PR 12w | 1b | 12.00 | 6.77 | 0.70 | 38.57 | 5.61 | 1 | 0.00 | 5.61 | 54 |
| PROVE1 | TVR/PR 12w + PR 12w | 1b | 12.00 | 6.66 | 0.70 | 17.14 | 6.60 | 4 | 33.14 | 6.77 | 54 |
| PROVE2 | TVR/PR 12w + PR 12w | 1b | 12.00 | 6.66 | 0.70 | 14.00 | 1.30 | 2 | 20.57 | 6.13 | None |
| PROVE2 | TVR/PR 12w + PR 12w | 1b | 12.00 | 6.72 | 0.70 | 13.86 | 1.30 | 4 | 10.00 | 6.48 | 36 |
| PROVE2 | TVR/PR 12w + PR 12w | 1b | 12.00 | 7.00 | 0.70 | 24.00 | 4.65 | 2 | 10.57 | 5.84 | 156 |
| PROVE2 | TVR/PR 12w + PR 12w | 1b | 11.86 | 6.02 | 0.70 | 19.57 | 3.53 | 1 | 0.00 | 3.53 | 54 |
| PROVE2 | TVR/PR 12w + PR 12w | 1b | 12.00 | 6.46 | 0.70 | 20.00 | 6.16 | 1 | 0.00 | 6.16 | 54 |
| PROVE2 | TVR/PR 12w + PR 12w | 1b | 12.00 | 6.21 | 0.70 | 20.57 | 4.91 | 1 | 0.00 | 4.91 | 54 |
| PROVE2 | TVR/PR 12w + PR 12w | 1b | 12.00 | 6.42 | 0.70 | 16.00 | 2.26 | 1 | 0.00 | 2.26 | 54 |
| PROVE1 | TVR/PR 12w + PR 12w | 1b | 12.00 | 6.71 | 0.70 | 50.43 | 2.55 | 2 | 1.71 | 6.15 | 54,156 |
| PROVE2 | TVR/PR 12w + PR 12w | 1b | 12.14 | 7.20 | 0.70 | 14.00 | 1.30 | 2 | 2.00 | 6.03 | 36 |
| PROVE2 | TVR/PR 12w + PR 36w | 1b | 12.00 | 6.48 | 0.70 | 34.14 | 3.16 | 1 | 0.00 | 3.16 | 54 |
| PROVE2 | TVR/PR 12w + PR 36w | 1b | 12.00 | 6.89 | 0.70 | 30.43 | 5.39 | 2 | 2.00 | 5.62 | 36 |
| PROVE1 | TVR/PR 12w + PR 36w | 1b | 11.86 | 5.92 | 0.70 | 38.00 | 3.01 | 2 | 3.00 | 4.82 | 36 |
| PROVE2 | TVR/PR 12w + PR 36w | 1b | 12.14 | 7.25 | 0.70 | 26.00 | 1.30 | 2 | 2.43 | 5.37 | 36 |
| PROVE2 | TVR/PR 12w + PR 36w | 1b | 12.14 | 5.67 | 0.70 | 29.00 | 5.45 | 1 | 0.00 | 5.45 | 36 |
| PROVE2 | TVR/PR 12w + PR 36w | 1b | 12.00 | 6.26 | 0.70 | 24.14 | 3.10 | 2 | 1.86 | 4.49 | 36,54 |
| PROVE1 | TVR/PR 12w + PR 36w | 1b | 12.00 | 7.03 | 0.70 | 41.57 | 5.95 | 2 | 4.00 | 5.65 | 54 |
| PROVE2 | TVR/PR 12w + PR 36w | 1b | 12.00 | 6.12 | 2.41 | 1.00 | 2.49 | 12 | 27.00 | 5.41 | 54,156 |
| PROVE2 | TVR/PR 12w + PR 36w | 1b | 11.86 | 7.20 | 0.70 | 31.86 | 3.17 | 2 | 4.71 | 5.56 | 156 |
| PROVE2 | TVR/PR 12w + PR 36w | 1b | 2.14 | 6.93 | 1.30 | 3.00 | 1.52 | 2 | 2.14 | 3.45 | 36 |
| PROVE2 | TVR/PR 12w + PR 36w | 1b | 12.00 | 6.72 | 0.70 | 33.57 | 6.00 | 1 | 0.00 | 6.00 | 156 |

*^1^, Treatment arm; P=Peg interferon alfa-2a, R=Ribavirin, w=weeks; dosing information after the ‘+’ symbol indicates PR dosing regimen after a dosing window of telaprevir and peginterferon alfa-2a plus or minus ribavirin. ‘None’ indicates that no PR window followed dosing window that included TVR.*

*^2^, Weeks on TVR*

*^3^, Log of the viral load at baseline*

*^4^, Log of the viral load at the nadir time-point after the start of dosing. Values of ‘1.30’ indicate that HCV RNA was detected but not quantifiable. Values of ‘0.70’ indicate that HCV was not detected at the nadir time-point*

*^5^, Time in weeks from the start of dosing to a post-nadir time-point where viral load is above nadir*

*^6^, Log of the viral load at the time-point defined in column superscripted by ‘4’*

*^7^, Number of available time-points after the time-point defined in the column superscripted by ‘4’*

*^8^, Time in weeks between the last available time-point and the time-point defined in the column superscripted by ‘4’*

*^9^, Log of the viral load at the last available time-point*

*^10^, Positions with TVR-resistance-associated mutations at at least one time-point post-treatment*

*^11^, Patient classified as neither Breakthrough nor Relapser. The first time-point with viral sequence data after baseline is used to indicate the post-nadir time-point*
